# Uncoupling shear and uniaxial elastic moduli of semiflexible biopolymer networks: compression-softening and stretch-stiffening

**DOI:** 10.1038/srep19270

**Published:** 2016-01-13

**Authors:** Anne S. G. van Oosten, Mahsa Vahabi, Albert J. Licup, Abhinav Sharma, Peter A. Galie, Fred C. MacKintosh, Paul A. Janmey

**Affiliations:** 1 Institute for Medicine and Engineering, University of Pennsylvania, Philadelphia, PA; 2 Department of Physics and Astronomy, VU University, 1081HV Amsterdam, the Netherlands; 3 Third Institute of Physics, Georg August Universität, 37077 Göttingen, Germany; 4 Department of Biomedical Engineering, Rowan University, Glassboro, NJ

## Abstract

Gels formed by semiflexible filaments such as most biopolymers exhibit non-linear behavior in their response to shear deformation, e.g., with a pronounced strain stiffening and negative normal stress. These negative normal stresses suggest that networks would collapse axially when subject to shear stress. This coupling of axial and shear deformations can have particularly important consequences for extracellular matrices and collagenous tissues. Although measurements of uniaxial moduli have been made on biopolymer gels, these have not directly been related to the shear response. Here, we report measurements and simulations of axial and shear stresses exerted by a range of hydrogels subjected to simultaneous uniaxial and shear strains. These studies show that, in contrast to volume-conserving linearly elastic hydrogels, the Young’s moduli of networks formed by the biopolymers are not proportional to their shear moduli and both shear and uniaxial moduli are strongly affected by even modest degrees of uniaxial strain.

Networks formed by filamentous biopolymers such as intracellular proteins like actin and vimentin, as well as extracellular proteins like collagen and fibrin, show distinct nonlinear viscoelastic mechanical responses when deformed in shear. The shear storage modulus *G′*, of such networks is higher than that of flexible polymer networks with the same mass density^1^. The storage modulus increases with concentration as 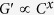, where *x* 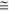 2 to 2.5 for both intracellular and extracellular networks^2^,^3^,^4^,^5^,^6^,^7^,^8^. The large elastic moduli and their strong dependence on polymer density occur even though biopolymer networks fall below the isostatic threshold. This threshold corresponds to a 6-fold connectivity for minimal mechanical stability of 3D networks with only central-force (i.e., stretching) interactions^9^. In most 3D biopolymer networks, the nodes consist of either two cross-linked fibre segments with coordination number z = 4, or branching points with z = 3. Thus, the stability of biopolymer networks must be due to other factors, such as the bending rigidity of fibres, or internal stresses such as those applied by motor proteins^10^,^11^,^12^,^13^.

Both biological and synthetic semiflexible polymer networks also show dramatic nonlinear elastic effects, including shear strain stiffening at relatively low shear strains, depending on network density and polymer stiffness^14^,^15^. Strain-stiffening can be understood either by the non-linear force extension relation of semiflexible polymers due to thermal undulations at the filament level, or by collective rearrangements and alignment of filaments at the network level^16^. When sheared, these networks also exhibit large negative axial (or normal) stress; in contrast, flexible elastomers exhibit a much smaller positive (compressive) normal stress. Therefore, biopolymer networks would tend to collapse upon applying shear, whereas linear elastic polymer networks would expand^17^. This effect might complicate measurement of shear moduli in stress-controlled shear rheometers that automatically alter plate spacing to maintain a constant (usually zero) axial stress.

Nearly all measurements of the non-linear rheology of semiflexible polymer networks have been done under shear deformation, with the assumption that the Young’s modulus is two to three times larger, depending on the Poisson’s ratio of the materials. Young’s moduli have been directly measured for collagen and fibrin gels, but not directly related to rheology under shear strain.

Interest in biopolymer mechanics has increased since it has been shown that mechanical properties of cell substrates or extracellular matrix (ECM), influence cell functions^18^. Changes of the ECM have been linked to, or even precede common pathologies such as cancer^19^, atherosclerosis^20^ and fibrosis^21^. Tissue engineering requires detailed knowledge of the mechanics of the materials used as scaffolds to replace tissues. For this purpose, reconstituted networks and tissues have been characterized with either shear or uniaxial testing methods. However, most tissues are subjected to multiaxial mechanical stimuli at a variety of time scales.

In the present work, we adapt common rheological techniques to provide a mechanical characterization of collagen and fibrin networks undergoing multiaxial deformations that mimic strains occurring *in vivo*. Rheological tests were done on networks with concentrations ranging from 2 mg/ml to 10 mg/ml, which spans the concentrations at which these biopolymers are found *in vivo*^22^.

We show that shear moduli decrease to an equilibrium value when networks are compressed but show a constant increase when samples are extended. Young’s moduli are significantly lower in compression compared to extension. When comparing apparent Young’s and shear moduli over a range of axial strains, the networks’ Young’s moduli are not linearly related to their shear moduli.

## Findings

**Figure 1:**
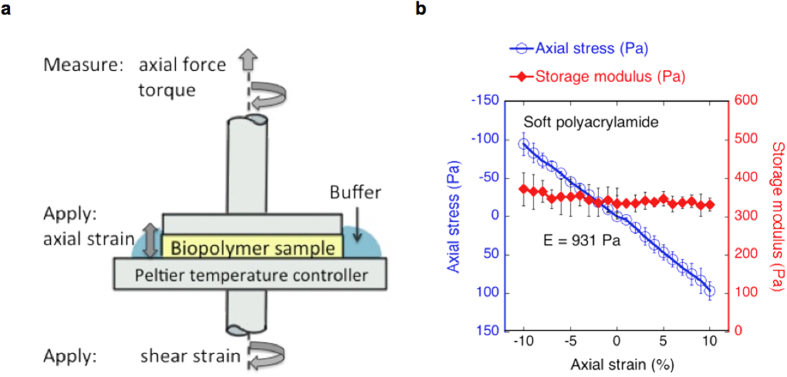
Experimental set-up and control experiments for multiaxial rheology method. (**a**) A shear rheometer is used with a parallel plate geometry. Biopolymer samples are polymerized between the plates; after polymerization buffer is added around the sample in order to prevent drying and allow free fluid flow into and out of the sample. The gap between the plates is changed to apply axial strain; the lower plate is rotated to apply shear strain. The torque and axial force are recorded. (**b**) The storage moduli measured with a shear rheometer and axial stress measured with a tensile tester of polyacrylamide gels (PAA). The PAA gel results show that for pure elastic linear materials the storage modulus is independent of the gap height and that the axial stress-strain curve is linear and symmetrical in compression and extension. The Young’s modulus is calculated from the slope of the stress-strain curve as *E* = *2.8* × *G′*.

Strain-stiffening and negative normal stress of semiflexible polymer gels suggest that shear moduli might be altered by internal stresses, generated by axial strain orthogonal to the shear plane. Figure 1a shows the experimental system that allows compression or extension to be applied to disk-shaped samples, while their shear modulus is measured by oscillatory shear displacements. A strain-controlled rheometer with parallel-plate geometry was used to apply axial strain by changing the gap after the network was fully formed. The parallel plate configuration results in a shear strain field that is zero in the centre and increases proportionally with the radius of the plate. A reservoir of solvent surrounds the sample to allow volume change by fluid flow across the free edges. As a control, linearly elastic polyacrylamide gels (PAA) were tested. These tests show that the storage moduli of PAA are independent of the level of axial strain. The axial stress is linear with axial strain (Fig. 1b). The Young’s modulus of PAA is *E* = *2.8* × *G′* over the entire range of deformations, and is in close agreement with the reported Poisson’s ratio of 0.486^23^.

**Figure 2:**
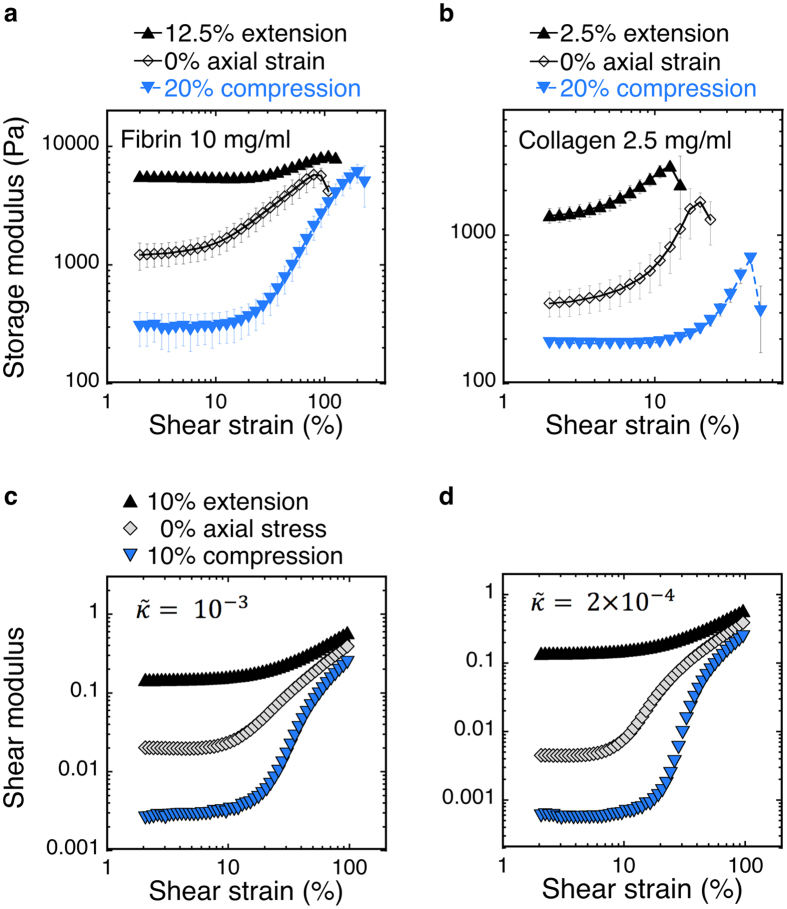
Shear strain stiffening of biopolymer networks shows alterations in onset and amplitude when compressed or extended. Experimental results for fibrin (**a**) and collagen (**b**); the mean of 3 samples is shown ± SD. Both biopolymer networks have a decreased storage modulus at low shear strains when compressed and an increased modulus when extended. For fibrin the storage modulus converges at high shear strains. (**c**,**d**) Storage modulus versus shear strain for a diluted phantomised triangular network with *L/l*_*c*_ = 6.67 (local coordination number z = 3.4); with varying pre-stress: without any axial strain, with 10% extension and with 10% compression. The normalised bending modulus 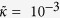 (**c**) corresponds with fibrin, 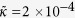 with collagen (**d**).

Networks of collagen at 2.5 mg/ml and fibrin at 10 mg/ml were subjected to increasing shear strain amplitudes after static compression or extension. An oscillatory shear strain of constant frequency and increasing magnitude was applied. The shear storage moduli were compared to strain-stiffening without axial strain (Fig. 2).

Collagen and fibrin networks stiffen with increasing shear strain, both when extended and compressed, although the storage modulus at low shear strains is altered dramatically by application of axial strain, it is lower in compression and larger in extension compared to uncompressed samples. For collagen, the onset of strain-stiffening occurs at larger shear strains under compression, and the critical strain at which strain-softening is observed is higher. Under extension, collagen networks show a similar onset of strain-stiffening, but the critical strain is lower compared to the samples without axial strain (Fig. 2b). In fibrin networks, the onset of strain-stiffening is higher under extension than under compression. However, the absolute peak values of *G′* are similar for the three levels of axial strain. (Fig. 2a)

The axial stress consists of both the negative contribution in response to the shear deformation and the applied axial stress. If the sample is extended, the axial stress is positive while a compressed sample shows negative axial stress. This sign convention is used for consistency with axial strain measurements.

To model networks of fibrin and collagen, we generated disordered, lattice-based networks such that the average coordination number (connectivity) z = 3.4 is consistent with direct observations of collagen networks^24^. In order to model networks with local connectivity of less than four, our 3D networks are generated by dilution of a *phantomised* FCC structure, in which the six fibers crossing at a node are separated randomly into three cross-linked pairs^25^, using freely-hinged joints. To reduce any edge effects, periodic boundaries are used for networks under deformation (see SI).

The network connectivity is well below the point of marginal stability for purely spring/stretching interactions^9^ suggesting that the stability (finite shear modulus) of such networks arises from additional stabilizing interactions, such as bending and applied stress. We model the total elastic energy 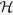 of the network by combining bending and stretching contributions of all the fibers *f*:


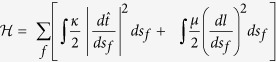


Here, *κ* is the bending rigidity of the individual filaments and *μ* is their stretch modulus (see SI). The dimensionless bending rigidity 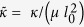, where *l*_*0*_ is the lattice spacing. In our 3D lattice-based networks, *l*_*0*_ is the same as *l*_*c*_, which is the spacing between adjacent crosslinkers.

We vary *κ*, keeping *μ* = *1* fixed. For collagen and fibrin fibers the dimensionless bending rigidity 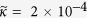 and 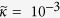, have been chosen respectively (see SI).

The linear shear modulus is defined as 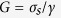
where *σ*_*s*_ is the shear stress and *γ* is the shear strain. The shear stress is calculated as 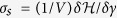 where *V* is the volume of the system. Axial stresses are calculated from the energy changes due to an infinitesimal axial deformation *δε* using fixed lateral boundaries.

The simulated networks were first compressed or extended before applying an increasing simple shear strain. This enables comparison with the experimental results shown in Fig. 2a,b. As a reference, we also show experiments and simulations without axial strain. Figure 2c,d show the results from 3D network simulations with parameters quantifying the filament rigidity and network mesh size comparable to experiments (see SI). As in the experimental results, applying axial stress changes the storage modulus.

**Figure 3:**
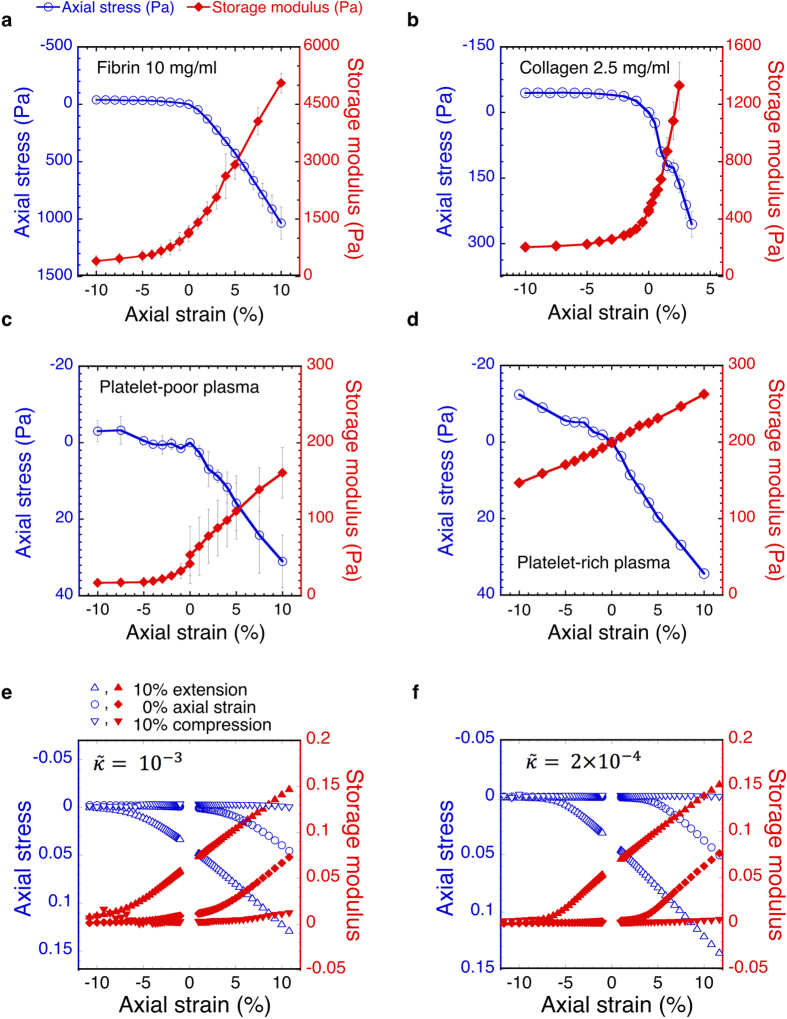
Axial stress and storage modulus of biopolymer networks as a function of axial strain. 10 mg/ml fibrin (**a**) 2.5 mg/ml collagen (**b**) platelet-poor plasma (**c**) and platelet-rich plasma (**d**) were subjected to a series of step-wise increases of the axial strain; samples were allowed to relax between subsequent steps. For fibrin and collagen extension and compression series were performed on separate samples; the mean of 3 samples is shown ± SD. For platelet-rich plasma (PRP) 1 exemplar is shown, which was compressed first, returned to 0% axial strain and subsequently extended. For platelet poor-plasma (PPP) the mean of 2 samples is shown ± SD. One sample was axially strained in the same manner as PRP; the second was extended first, returned to 0% and subsequently compressed. For all biopolymer networks the storage modulus decreases with compression, and increases with extension. For collagen, fibrin and PPP this response is asymmetric; for PRP the response is symmetric. The axial stress response is equally asymmetric for collagen, fibrin and PPP; whereas PRP shows a less asymmetric response. Simulation for a diluted phantomised triangular network (3D) with varying pre-stress and *L/l*_*c*_ = 6.67 (local coordination number z = 3.4) (**e**,**f**) shows the same trend for axial stress and storage modulus. The normalised bending modulus 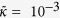 (**e**) corresponds with fibrin, 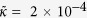 with collagen (**f**).

The effects of axial strain on the shear storage moduli of fibrin and collagen networks are shown in more detail in Fig. 3. Networks were subjected to an incremental series of compressions or extensions, while simultaneously measuring the dynamic shear moduli at an amplitude of 2% shear strain and frequency of 10 rad/s. Between steps, the networks were allowed to relax for a duration of 100 to 1200 seconds (depending on step size and sample) before applying the next step of axial deformation. The relaxed values of the storage moduli were plotted for every level of axial strain (Figure S1).

The storage moduli in the absence of axial strain were 1138 Pa ± 160 Pa (SD) and 458 Pa ± 23 Pa (SD) for the fibrin and collagen, respectively. Both storage moduli increase steadily when extended, with collagen networks stiffening faster than fibrin. In compression, the storage modulus shows a decrease, which levels out between 5–10% compression (Fig. 3a,b). At compression levels higher than 10% the storage modulus becomes constant, within the error of measurement (Figure S2).

The loss tangent (i.e., the ratio of loss to storage modulus) of 10 mg/ml fibrin is 0.022 ± 0.006 (SD) and for 2.5 mg/ml collagen it is 0.064 ± 0.04 (SD). When compressed, the loss tangent increases slightly and when extended it decreases, although overall the loss tangent remains small. (Figure S3). The experiments with fibrin networks were repeated with networks at 2 mg/ml, the results of which are analogous to the results with 10 mg/ml fibrin networks (Figure S4).

A tensile tester was used to measure the axial stress after relaxation for similar levels of axial strain, as were used with the shear rheometer. From this, axial forces can be measured more accurately than with the shear rheometer. The slope of the axial stress-strain curve defines an apparent Young’s modulus. Extension is defined as a positive axial strain whereas compression is a negative axial strain. In order to attain a positive slope, a positive axial stress is defined as the sample pulling on the upper plate in extension.

Collagen and fibrin networks show a similar trend. In extension, the axial stress increases approximately linearly with the axial strain over the entire range. By contrast, the stress-strain relationship is nonlinear up to ~4% compression, with a significant reduction in the slope. At larger compression, the response becomes linear. (Fig. 3a,b). We determine the Young’s modulus in this linear region, between 4% and 10% compression, where it is much smaller than what we measure under extension. For collagen, the apparent Young’s modulus in compression is 29.1 Pa and 6.52 kPa in extension. For fibrin in compression it is 128 Pa in compression, and 10.7 kPa in extension. The ratio of the apparent *E* in extension to the apparent *E* in compression is 224 for collagen and 84 for fibrin.

The simulated networks were subjected to increments of compression or extension, while measuring the linear shear modulus (at shear strains of 1%). At each step, the network is allowed to relax; the energy is minimized before applying the next axial strain step. The simulation results in Fig. 3e,f agree well with the measured dependence of *G′* on axial strain as seen in Fig. 3a,b. Our model can account for the features observed in the experiments, including the qualitative differences between collagen and fibrin, e.g., the sharper onset of stiffening with axial strain.

The strong effects of external stress on the shear modulus of these networks suggest that generation of internal stress within the network, such as those generated *in vivo*, would alter the effects of axial strain on the shear modulus. To determine the effect of internal stress on fibrin networks, blood plasma containing fibrinogen as its major polymerizing protein was prepared with and without platelets. These cells bind the fibrin strands in a clot formed after activation of fibrinogen by thrombin and put them under tension^26^. Platelet-rich (PRP) and platelet-poor plasma (PPP) clots were subjected to similar multiaxial tests. Because of the rigid adhesive boundaries the parallel plates provide, the fibrin network is put under significant stress. This is confirmed by the development of a significant axial stress of 46 Pa for PRP (n = 1), whereas the axial stress in PPP is positive but small, on the order of single Pa. The platelet contractility causes the moduli of the PRP to increase drastically. Also, the response of the shear modulus is now symmetric over the tested range of axial strains; the axial stress shows less asymmetry between compression and extension compared to the unstressed networks. PPP clots show very similar behaviour as collagen and fibrin networks. (Fig. 3c,d)

Figure 3e,f show simulations of networks generated in the absence of pre-stress, and with an imposed pre-stress equivalent to 10% extension and 10% compression. Both the storage moduli and axial stresses show a 10% shift in their response. The compressed networks stiffen later, the extended networks stiffen earlier. As a result the extended curves show a symmetric response around 0% axial strain.

In contrast to polyacrylamide gels, the relationship between the storage and Young’s moduli of fibrin and collagen gels varies with the axial strain. In extension the Young’s modulus stays constant within the tested range, whereas the storage modulus continues to increase. When compressed, both the storage and Young’s moduli approach new limiting values at large strains. However, between 4% and 10% compression the shear storage modulus of collagen is an order of magnitude larger than the Young’s modulus. This result means that the resistance of the network to compression is limited, while having a higher resistance to shear deformation.

## Discussion

Polymer networks in soft biological materials are subjected to simultaneous axial and shear deformation. For example, blood vessels are subjected to shear strain from fluid flow and extensional and compressive strains from dilation and constriction. Adipose tissue is statically sheared and compressed at long intervals when sitting.

Analysis of such materials generally assumes a simple relationship between the resistance to axial compression and extension, defined by the Young’s modulus *E*, and the resistance to shear deformation, defined by the shear modulus *G′*. For elastic solids, these quantities are generally related by *E* = *2G(1* + *ν)*, where *ν* is the Poisson’s ratio: the ratio of transverse strain to axial strain, which also quantifies the extent to which the sample maintains volume when it is compressed. Nearly all simple materials have Poisson’s ratios of 0 < *ν* < 0.5, meaning that *2G* < *E* < *3G*, where the upper limit is for incompressible systems. This assumption underlies all measurements of elastic moduli by atomic force microscopes or other indentation probes, which produce strains that are combinations of simple shear and uniaxial compression as well as extension.

The experiments reported here show that the elastic shear moduli of collagen and fibrin networks decrease under compression and increase when samples are extended. The Young’s moduli are an order of magnitude lower in compression compared to extension. The change of the elastic shear modulus is decoupled from the change of the Young’s modulus during compression and extension: in extension the Young’s modulus is nearly constant, whereas the shear modulus continues to increase. Furthermore, when networks are compressed by only a small percentage of the original height, the Young’s modulus drops below the shear modulus.

When subjecting the samples to increasing shear strain under axial deformation, the strain-stiffening behaviour is altered both in onset and amplitude. There are subtle differences between the response of fibrin and collagen when the networks are subjected to an increasing shear strain after axial stress application. For example, the critical strain shift is more pronounced for collagen than fibrin, which is not seen in the simulations. Overall, the simulations capture the behaviour of the networks well, but several assumptions are made that might be more applicable to collagen or fibrin. Also, the level of applied extension differs between collagen and fibrin, which could contribute for the differences observed.

The structures of fibrin and collagen differ, especially at the single fibre level. In the reconstituted collagen networks, there is no enzymatic cross-linking whereas the fibrinogen monomers within fibres are cross-linked with factor XIIIa (Figure S7). Moreover, the ability of single fibrin fibres to increase in length, after becoming straight, due the monomer stretching and sliding, is much greater than that of collagen fibres. In the simulations the fibres are considered to be a constant length (no stretching of individual fibres once fully straightened). At the network level, the nodes of collagen are entangled, whereas in fibrin networks branch points form, and loose fibre ends are rarely found in a fibrin network.

While collagen and fibrin have different structures in detail, there are reasons to apply the same model to them. Prior studies of similar models have shown a (perhaps surprising) degree of insensitivity to network structure in the predicted mechanics. For instance, Licup *et al.* demonstrated very close correspondence in the form of the nonlinear mechanics of lattice-based networks in both 2D and 3D, as well as so-called Mikado models of entirely randomly placed and cross-linked rods^27^.

The increase in storage moduli of fibrin networks due to active pre-stress generation has been described before using conventional shear rheometry^26^,^28^,^29^. The multiaxial rheology of pre-stressed networks reveals several interesting features. Even though the networks still compression-soften and extension-stiffen, the symmetry of the response is greatly enhanced. The simulations reveal that a 10% extensional pre-stress results in a symmetric response from 7% compression up through the whole range of extension. The internal pre-stress can be estimated in the experimental system from the development of axial stress during polymerization, while the gap is kept at a constant height. For PRP with an elastic shear modulus of 199 Pa the axial stress that develops during polymerization is 46 Pa, which is 23% of the initial shear modulus. Although this number cannot be compared directly to the amount of pre-stress used in the simulations, it indicates that there is a higher pre-stress than 10%. It is therefore likely that the symmetry seen over the whole test range for PRP, is due to the higher pre-stress imposed on the network.

The simulations show a simple shift in the response of the network to axial strain with imposed pre-stress. This is not seen when comparing the non-stressed networks collagen, fibrin, and PPP, to PRP. However, there are several parameters that could contribute to these differences. The platelets not only put tension on the networks, they also aggregate and form additional cross-links in the network^30^. PPP, fibrin and collagen are entangled and branch (in the case of fibrin and PPP). Blood proteins other than fibrin like albumin, globulins and fibronectin are present in plasma and are known to influence fibrin structure and mechanics^31^.

It is important to note that in the experiments described here, the samples are completely surrounded by buffer, and fluid is allowed to flow freely in and out of the network when axially strained, allowing the sample volume to change. This change was visually observed by photographing the boundaries of the gels after axial strain, which remain the same for collagen and fibrin. Thus, our results are consistent with a vanishing Poisson’s ratio. Here, the Young’s modulus coincides with the longitudinal modulus, in which the lateral dimensions of the sample do not change. For this reason, we impose fixed lateral boundaries in our simulations (see SI). The in/outflow of fluid and resulting compressibility of collagen has been described previously^32^. We also verified the water flow out of a collagen network by incorporating dye in a collagen network and measuring the dye in the surrounding buffer after compression as a function of time. (see SI and figure S5) By contrast, in our experiments on PAA gels, we observe boundaries that bulge under axial compression and are concave in extension, consistent with the near incompressibility of PAA which has a mesh size orders of magnitude lower than than of fibrin or collagen.

The volume change of collagen results in higher (lower) protein concentrations in compression (extension). The changes in the storage modulus are opposite to what would be expected from the change in polymer concentration due to the change in volume. The increased polymer mass in compression and its dilution in extension make the effects of axial strain on storage moduli even more striking.

The few studies directly comparing mechanical responses of soft hydrogels after deformation in different directions show results consistent with our findings. In one study the compressive moduli of collagen gels measured with atomic force microscopy are between 2.85 Pa and 23.1 Pa, and the shear moduli measured with a rheometer are between 19.9 Pa and 152 Pa respectively, for collagen isolated from rats of increasing age^33^. Another study compared creep in confined compression and shear and concluded that the shear modulus was higher in shear than in compression^34^. It has been shown for fibrin gels that when compressed the shear modulus drops initially. The same study also imaged the networks during compression and showed directly that filaments buckle when compressed^35^.

**Figure 4:**
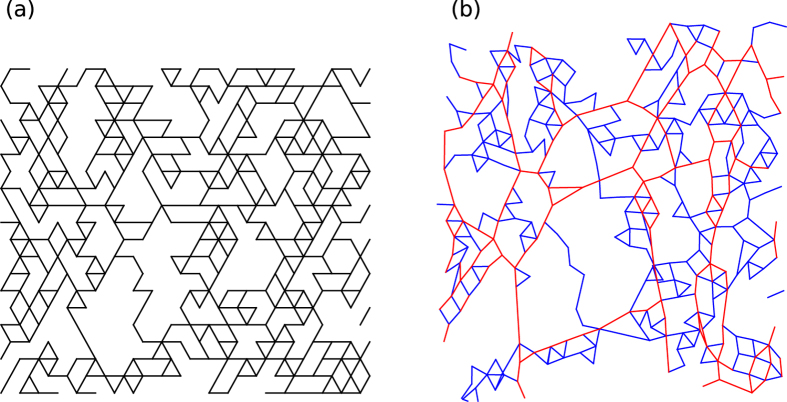
Representation of a relaxed and expanded network. A section of a 2D diluted triangular lattice with average coordination number z = 2.86. A relaxed configuration is shown in (**a**) in (**b**) the section has been uniaxially extended. The red coloring indicates segments under tension^40^.

Overall the following mechanism is proposed: As a sample is compressed it introduces a higher level of undulations and buckling, these individual buckled fibres do not contribute to the network stability which will result in a lower shear modulus and an inability to withstand axial loads. Extension removes undulations and will result in a higher shear modulus. The asymmetry seen in the response to compression and extension is caused by the bending contribution of the individual fibres being much lower than the stretching (straightening) contribution. In other words, it is easier to compress or buckle a fibre than it is to remove undulations from a fibre. (Fig. 4 and S6)

The relation between *E* and *G* in viscoelastic solids has previously been described to be extremely time dependent, with a Poisson’s ratio ranging from −1 < *ν* < 0.3^36^,^37^.

However, in our experiments, the networks are allowed to relax considerably and show even larger variability in the relationship between *E* and *G*. Thus, the decoupling of *E* and *G* is not merely a time dependence issue but an intrinsic material property. Fluid flow into the gel might account for the differences observed in previous studies, where extension of these gels is often described as viscoelastic. A logical continuation on the research presented in this paper is to analyse the time dependency of these systems.

Fibrin and collagen gels have often been suggested as scaffolds for cells in tissue engineering purposes. The mechanical adequacy has been often debated due to the variability in reported moduli. In this study it becomes apparent that the ability of these networks to withstand compression and the combination of shear and compression is limited, which might pose a problem when these gels are used to replace damaged tissues. It is vital to further study the mechanics of whole tissues and cell seeded biopolymer networks to deduct if this will improve the ability of constructs to resist to mechanical loads.

## Methods

### Preparation of fibrin

Fibrinogen (Fbg), isolated from human plasma and plasminogen depleted, (CalBioChem, EMD Millipore, Billerica, MA, USA) was dissolved in 1X T7 buffer (50 mM Tris, 150 mM NaCl at pH 7.4). Thrombin (Thr) isolated from salmon plasma (SeaRun Holdings, Freeport, ME, USA) was diluted in ddH_2_O at 1000 U/ml. Solutions were aliquoted and snap frozen for future use. Salmon thrombin clotting properties for human fibrinogen were checked previously^38^,^39^ and were found to be near identical to human thrombin at the Fbg and Thr concentrations used in this study. Factor XIII cross-linking was checked with a SDS PAGE gel and found identical between human and salmon thrombin (Figure S7).

To prepare fibrin networks, solutions were warmed to room temperature; fibrinogen stock solution, 1X T7 buffer, CaCl_2_ stock and thrombin were added at appropriate ratios to yield 10 mg/ml fibrinogen, 30 mM Ca^2+^ and 0.5 U thrombin/mg Fbg. The samples were polymerized at 25 °C.

### Preparation of collagen type 1

Collagen type 1 isolated from calf skin (MP Biomedicals, Santa Ana, CA, USA) was dissolved in 0.02N acetic acid. To prepare collagen networks 10X PBS, 0.1M NaOH and ddH_2_O were warmed to room temperature and added in appropriate ratios to yield a 2.5 mg/ml collagen concentration in 1X PBS solution with a pH between 7–7.5. The samples were polymerized at 37 °C.

### Preparation of platelet-rich and platelet-poor plasma

A blood donation was conducted in accordance with all appropriate guidelines and regulations and with approval of the Internal Review Board at the University of Pennsylvania (protocol nr. 805305). Human blood was drawn with informed consent from a healthy volunteer.

Whole blood was drawn via venipuncture from in K_3_EDTA. To obtain platelet-rich plasma the whole blood was centrifuged for 15 minutes at 120 × g, from which the supernatant was removed and used. Platelet-poor plasma was prepared by spinning whole blood at 2200 × g for 15 minutes. To prepare plasma clots salmon thrombin and calcium were added to yield 2U thrombin/ml and 30 mM Ca^2+^. The samples were polymerized at 37 °C.

### Preparation of soft polyacrylamide gels

40% acrylamide and 2% bis-acrylamide solutions (Bio-Rad, Hercules, CA, USA) were mixed with ddH_2_O to yield a 7.5% acrylamide and 0.01% bis-acrylamide solution. Polymerization was initiated by adding ammonium persulfate (APS) and tetramethylethylenediamine (TEMED). Gels were polymerized at room temperature in a well, overlaid with ddH_2_O, and cut into shape with a circular punch prior to measurements.

### Rheometry

A strain-controlled rotational rheometer (RFS3, TA Instruments, New Castle, DE, USA) was used with a parallel plate of 8 mm for 10 mg/ml fibrin; 25 mm diameter for 2.5 mg/ml collagen, both with a gap of 1 mm. PRP and PPP clots were measured with a 50 mm diameter plate with a 400 μm gap. The bottom plate incorporated a Peltier plate, allowing to control the sample temperature, 25 °C for 10 mg/ml fibrin and 37 °C for the other samples. The biopolymer samples were pipetted between the plates prior to polymerization. After polymerization, the appropriate buffer was pipetted around the free edge of the sample to prevent drying and allow free fluid flow in and out of the sample.

The shear moduli of the samples were measured by applying a low oscillatory shear strain of 2% at a frequency of 10 rad/sec. Axial strain was applied by changing the gap between the plates. See Fig. 1 for a schematic representation of the experimental set-up.

Some samples were subjected to a fixed step compression or extension after which a shear strain sweep was performed. Strain sweeps were performed in absence of axial strain and after applying 20% compression, 12.5% extension (for fibrin) and 2.5% extension (for collagen). The shear strain was increased from 2% up to the point of breakage, which depends on the sample type and the level of axial strain, at a frequency of 1 rad/sec. This lower frequency was necessary to observe the response of the axial stress to the applied shear stress.

Other biopolymer samples were subjected to either an incremental compression or extension series. The step-size was optimized for axial strain in each direction, separately for collagen, fibrin and plasma, to yield optimal resolution and prevent the sample from tearing (in extension). During the axial strain series the samples were allowed to relax between 100 and 1200 seconds before continuing to the next level of axial strain. For a precise application of axial strain the plate was moved at a low speed of 2 μm/s.

### Tensile testing

To obtain additional data on the mechanical properties of collagen and fibrin networks under axial deformation, a tensile tester was used (Instron 5564, Norwood, MA, USA), parallel platens were used at a gap of 1 mm, using similar volumes of fluid as used for rheometry.

To obtain data on fully relaxed networks; samples were subjected to 10 μm steps of compression or extension at 2 μm/s, which were allowed to relax for 15 minutes between consecutive steps.

## Additional Information

**How to cite this article**: van Oosten, A. S. G. *et al.* Uncoupling shear and uniaxial elastic moduli of semiflexible polymer networks: compression-softening and stretch-stiffening. *Sci. Rep.* **6**, 19270; doi: 10.1038/srep19270 (2016).

## Supplementary Material

Supplementary Information
