# Supplementary material for: Uncoupling shear and uniaxial elastic moduli of semiflexible biopolymer networks: compression-softening and stretch-stiffening
Source: Sci Rep. 2016 Jan 13;6:19270. doi: 10.1038/srep19270 (PMC4725936; doi:10.1038/srep19270)
Supplement: Supplementary Information [file srep19270-s1.pdf]

Supplemental document for:

Uncoupling shear and uniaxial elastic moduli of semiflexible biopolymer networks: compression-softening and stretch-stiffening

Anne S. G. van Oosten[1], Mahsa Vahabi[2], Albert J. Licup[2], Abhinav Sharma [2,3], Peter A. Galie[1,4] , Fred C. MacKintosh[2], Paul A. Janmey[1,*]

1. Institute for Medicine and Engineering, University of Pennsylvania, Philadelphia, PA
2. Department of Physics and Astronomy, VU University, 1081HV Amsterdam, the Netherlands
3. Third Institute of Physics, Georg August Universität, 37077 Göttingen, Germany
4. Department of Biomedical Engineering, Rowan University, Glassboro, NJ

Corresponding Author:

Paul A Janmey
Institute for Medicine and Engineering
1010 Vagelos Laboratories
3340 Smith Walk
Philadelphia, PA 19104
janmey@mail.med.upenn.edu
Phone: (215) 573-7380
Fax: (215) 573-6815

Supplemental figures

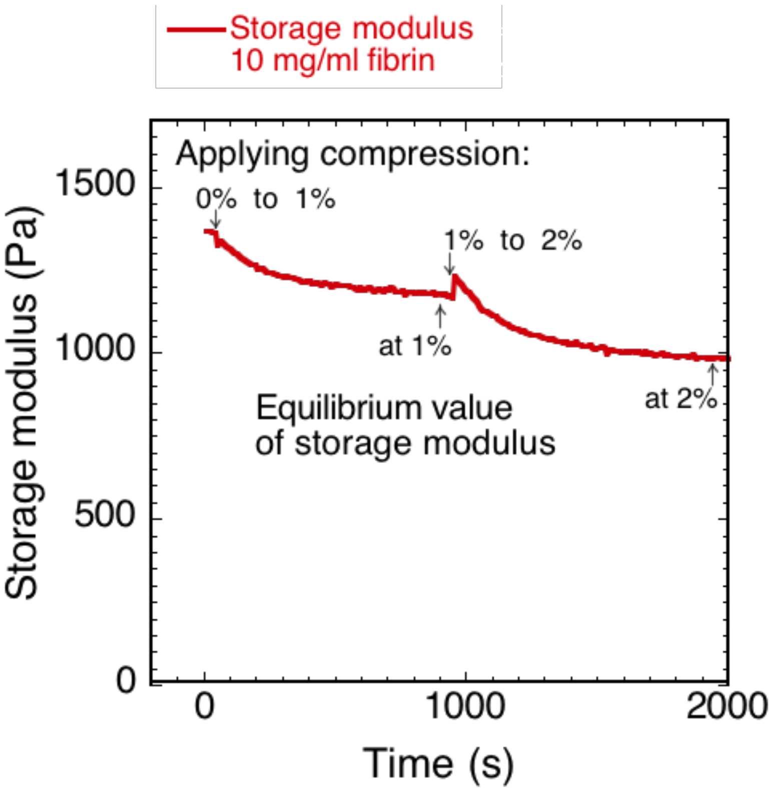

Figure S1 I Example of storage modulus of fibrin network followed in time during typical compression sequence. The storage modulus of a 10 mg/ml fibrin network is shown as a function of time during compression from 0% to 1% and from 1% to 2%. The 'equilibrium value' of the storage modulus is taken right before the next level of compression is applied, as pointed out by the arrows. The same approach is used for the storage modulus in extension and the axial stress.

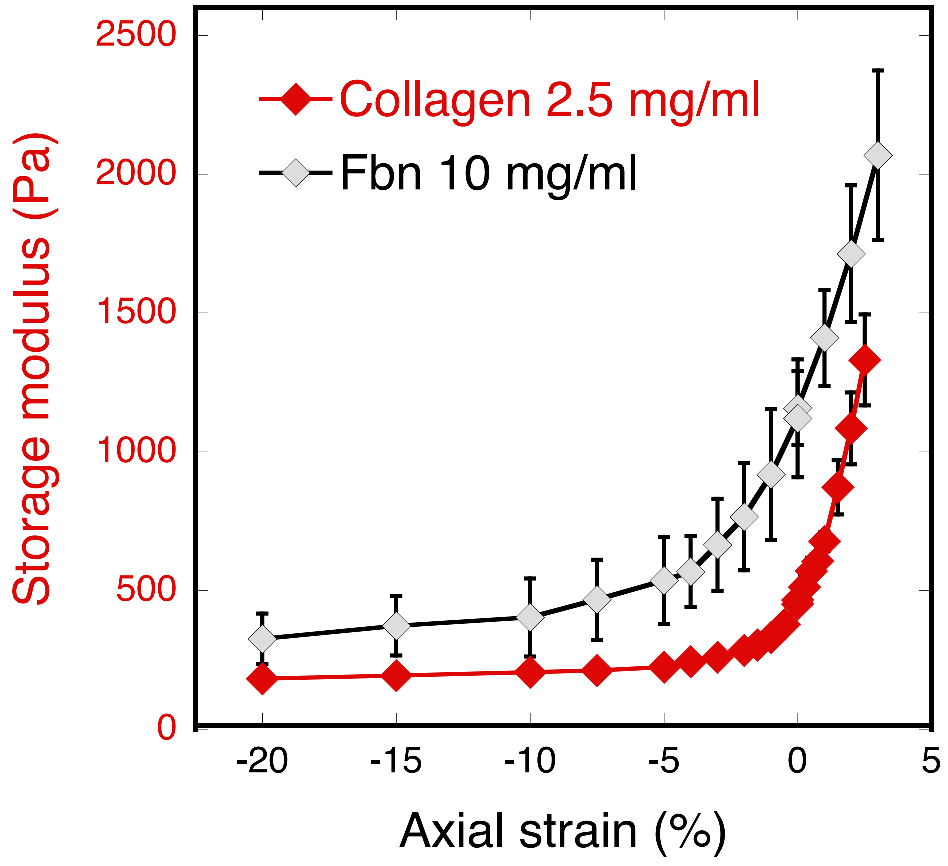

Figure S2 l Storage modulus of biopolymer networks as a function of axial strain. 10 mg/ml fibrin (open diamonds), 2.5 mg/ml collagen (red diamonds). The storage modulus is shown over a range of 20% compression (-20% axial strain) through 3% extension. The data between -10% and 3% are the same as shown in figure 2a and 2b of main article. The compression was continued up to 20% which shows that G' levels off between 10 and 20% compression within the error of measurement. The mean of 3 samples is shown ± SD

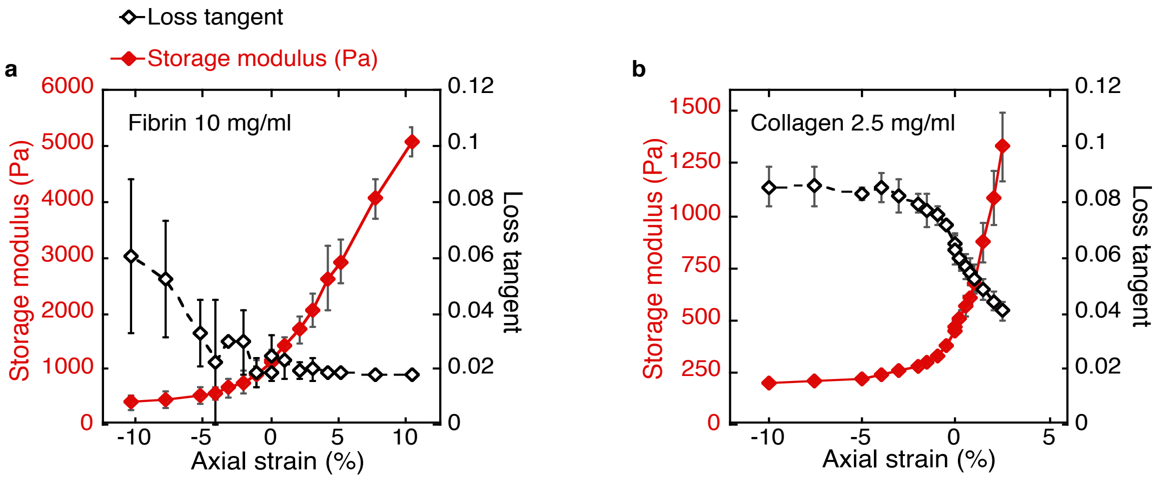

Figure S3 l Storage modulus and loss tangent of biopolymer networks as a function of axial strain. 10 mg/ml fibrin (**a**), 2.5 mg/ml collagen (**b**)
Storage modulus and loss tangent plotted as a function of axial strain for 10 mg/ml fibrin (**a**), 2.5 mg/ml collagen (**b**) The mean of 3 samples is shown ± SD

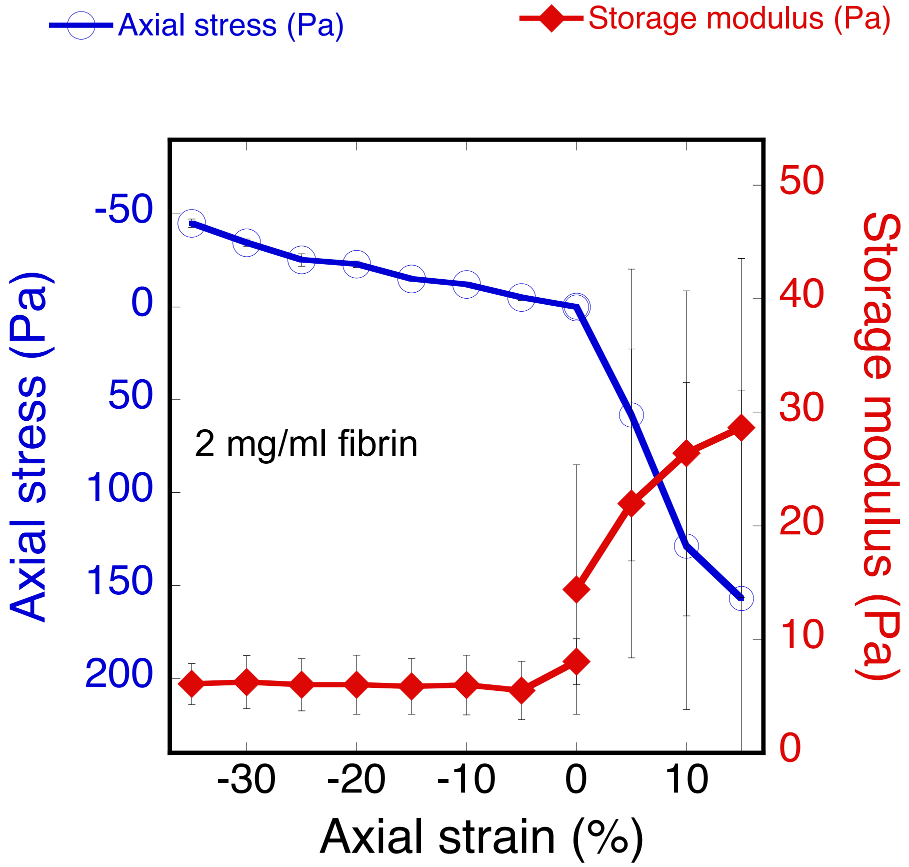

Figure S4 I Axial stress and storage modulus of biopolymer networks as a function of axial strain of 2 mg/ml fibrin network. The lower fibrin concentration shows similar response to axial strain as the 10 mg/ml fibrin network. Experimental protocol differed from 10 mg/ml fibrin as follows: 2 mg/ml Fibrin networks were tested at 37°C with a 50 mm plate with a gap of 400 µm. The axial stress was collected with the shear rheometer.

The samples were subjected first to a compression series up to 35%, after which the sample was slowly decompressed, allowed to equilibrate and subsequently extended. The mean of 3 samples is shown ± SD

Note: the storage modulus at the start of the extension series –after decompression- is higher than at the beginning of the compression series.

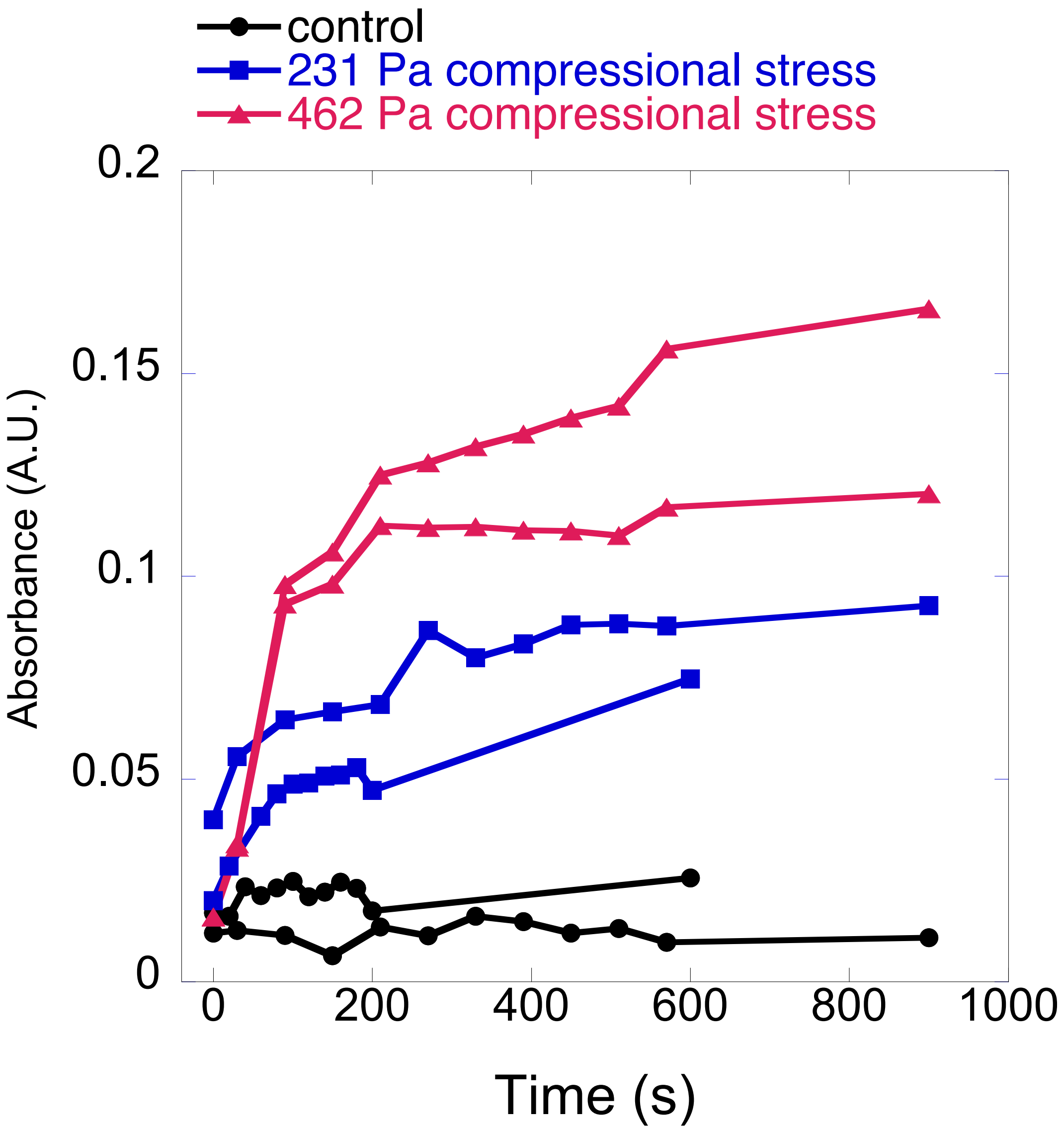

Figure S5 I Measuring dye out flux in buffer after compression. 2.5 mg/ml collagen with food colouring dye was compressed at 0, 231 or 462 Pa compressional stress. The influx of dye into the surrounding buffer was measured. Without compression the dye remains inside the gel, while compressive stress causes outflow of dye, which increases with increasing compression.

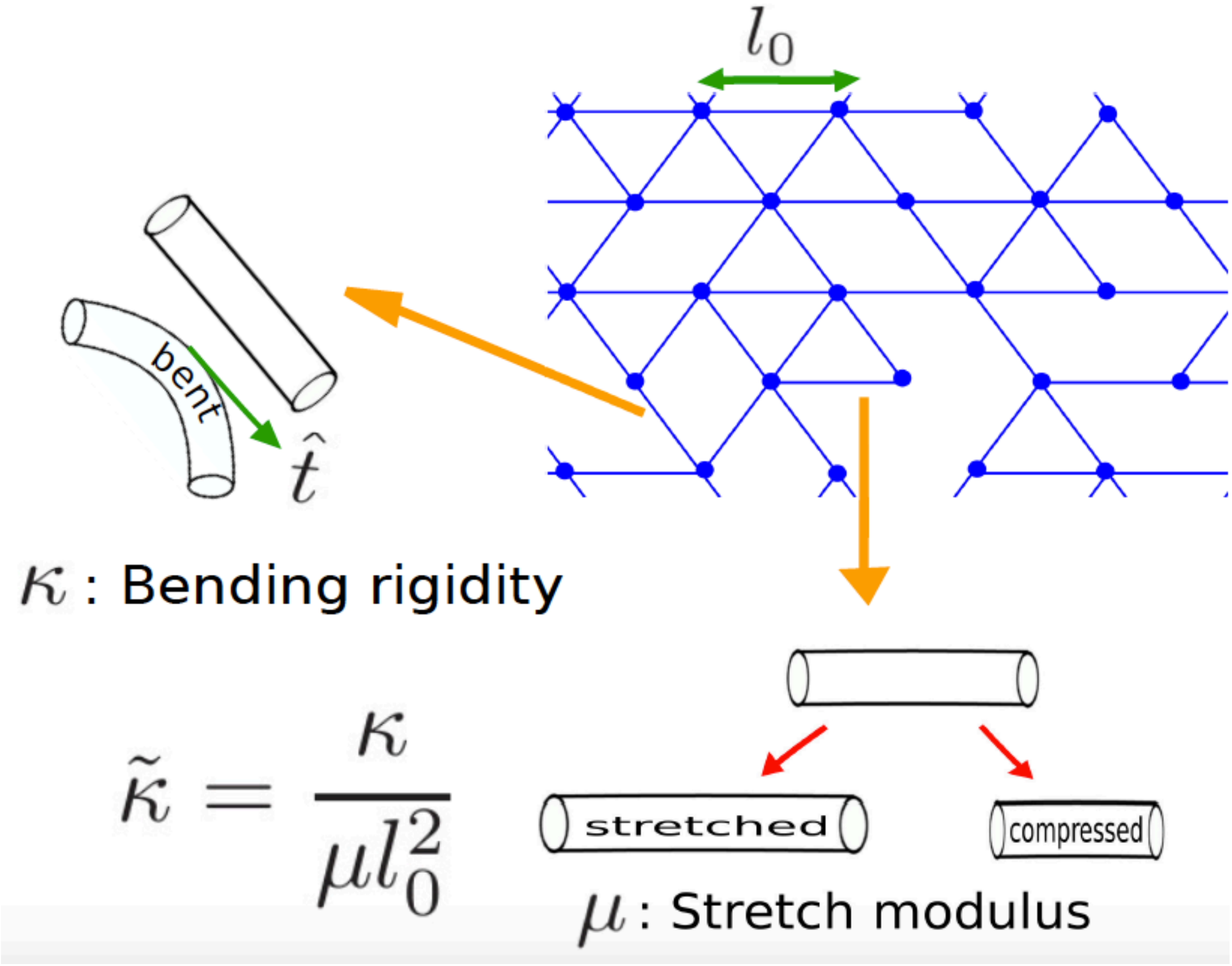

Figure S6 I 2D representation of diluted triangular lattice with explanation of model parameters.

1) Fibrinogen

2) Fbg + HuThr

3) Fbg + HuThr + 10mM Ca^{2+}

4) Fbg + HuThr + 30mM Ca^{2+}

5) Fbg + HuThr + DMEM

6) Fbg + SaThr

7) Fbg + SaThr + 10mM Ca^{2+}

8) Fbg + SaThr + 30mM Ca^{2+}

9) Fbg + SaThr + DMEM

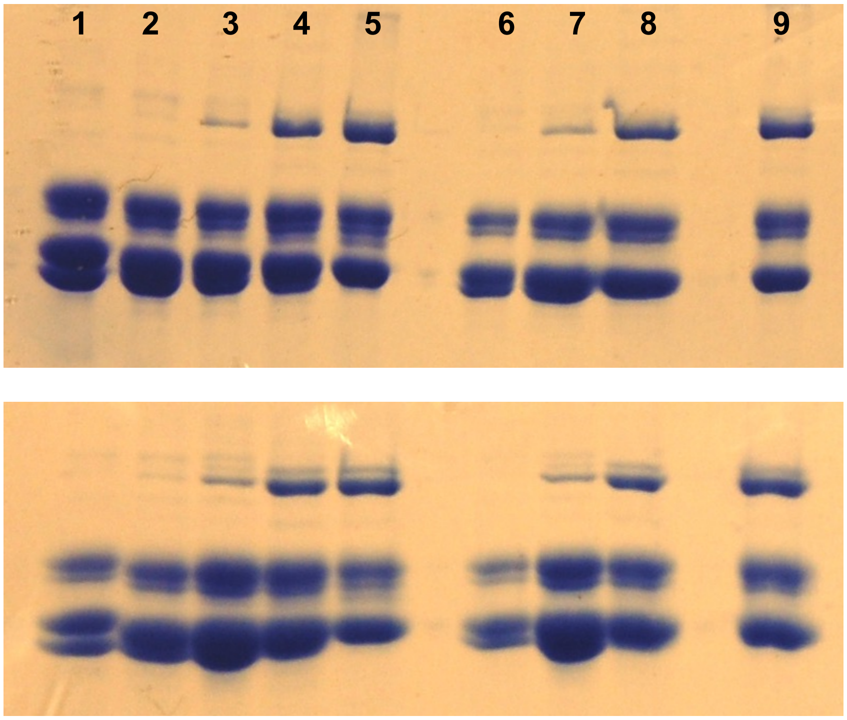

Figure S7: Cross-linking of fibrin by salmon thrombin. Human Fibrinogen from CalBioChem (Fbg) diluted to final concentration of 1 mg/ml in 1X T7 buffer. Cross-linked with human thrombin from Sigma (HuThr), or with salmon Thrombin from SeaRun (SaThr) final concentration 2 U/ml, buffer contained 10 mM or 30 mM Ca^{2+}. Certain samples were prepared with 1X DMEM cell culture media instead of T7 buffer. 2 separate experiments are shown.

Supplemental Information

Fibrin cross-linking by salmon thrombin tested with SDS-PAGE gel

Measuring fluid flow out of gel during compression

Collagen gels were prepared in the same manner as the rheology experiments, with the exception of incorporating blue food coloring to measure fluid transport during compression. Gels were polymerized in a well plate underneath an 18 mm diameter circular coverslip to constrain the gel diameter and bathed in 1X PBS. To compress the gels, defined masses were placed on top of the gel to exert a 231 Pa or 462 Pa compressional stress. At distinct time points, 100 μL was removed from the buffer. The absorption of dye in the buffer was measured using a spectrophotmeter and plotted as a function of time. The absorption of dye measured accounted for the decreasing amount of buffer in the well. As a control, the same experiment was performed without adding additional weight on the gels.

Simulations

The networks that are generated here are 3D diluted phantomised lattices. It is generated by modifying the diluted FCC lattices such that at every lattice vertex, one frees up a segment, which essentially allows it to move freely through the remaining cross-linked segments as a phantom chain. Periodic boundaries are imposed to reduce any edge effects. Lees-Edwards boundary conditions are used to calculate lengths of

fibers for networks under deformation[1]. We treat intersections of fiber segments as freely-hinged permanent crosslinks.

We model the total elastic energy $\mathcal{H}$ of the network by combining bending and stretching contributions of all the fibers f:

$$\mathcal{H} = \sum_f \left[\int \frac{\kappa}{2} \left| \frac{d\hat{t}}{ds_f} \right|^2 ds_f + \int \frac{\mu}{2} \left(\frac{dl}{ds_f} \right)^2 ds_f \right]$$

Here, κ is the bending rigidity of the individual filaments and μ is their stretch modulus, $d\hat{t}$ and dl are the unit tangent and longitudinal strain respectively at a point s_f along the fiber contour.

The dimensionless bending rigidity is defined by $\tilde{\kappa} = \kappa/(\mu l_0^2)$, which we vary in our networks while keeping $\mu = 1$ fixed. Here l_0 is taken to be the lattice spacing and average distance between adjacent cross-linkers. Given a homogeneous cylindrical rod of radius *r* and Young's modulus *E*, from classical beam theory[2], $\mu = \pi r^2 E$ and $\kappa = \frac{\pi}{4} r^4 E$. From this, $\tilde{\kappa} = \kappa/(\mu l_0^2) = r^2/(4 l_0^2)$, which is smaller than but proportional to the volume fraction ϕ of fibers in the network, since their cross-sectional area is πr^2 and there is a total length of order l_0 in a lattice/mesh volume of order l_0^3 [3]. For our collagen networks with concentration 2.5 mg/ml, the volume fraction $\phi \cong 0.2\%$. We thus use a smaller value of $\tilde{\kappa} = 2\times10^{-4}$ to compare with our collagen experiments. For the 10mg/ml fibrin networks, we use $\tilde{\kappa} = 10^{-3}$.

It is important to mention that normal stresses measured experimentally have an offset, which is set to zero in all the figures. This suggests doing simulations on networks which are initially prestressed. In Fig. 3e, 3f (theory), normal stress and shear modulus are obtained for diluted phantomised lattices (3D). The results from the lattice with no prestress or with prestress are compared. It can be clearly seen how prestress delays (compression cases) or hastens (extension cases) the stiffening.

References:

1. Lees, A. W., and S. F. Edwards. "The computer study of transport processes under extreme conditions." *Journal of Physics C: Solid State Physics* **5**, 1921 (1972)

2. Landau, L.D. & Lifshitz, E.M. *Theory of elasticity*, 2nd ed., Ch. 2 (Pergamon Press, Oxford, 1970).

3. Conti E. & F.C. MacKintosh, *Crosslinked networks of stiff polymers exhibit negative normal stress. Physical Review Letters* **102**, 088102 (2009).
